# Supplementary material for: The causal relationship between the human gut microbiota and pyogenic arthritis: a Mendelian randomization study
Source: Front Cell Infect Microbiol. 2024 Nov 26;14:1452480. doi: 10.3389/fcimb.2024.1452480 (PMC11629706; doi:10.3389/fcimb.2024.1452480)
Supplement: Supplementary file 1 [file DataSheet1.zip › mendelian test/ebi-a-GCST90027561.csv_leave_one_out.pdf]

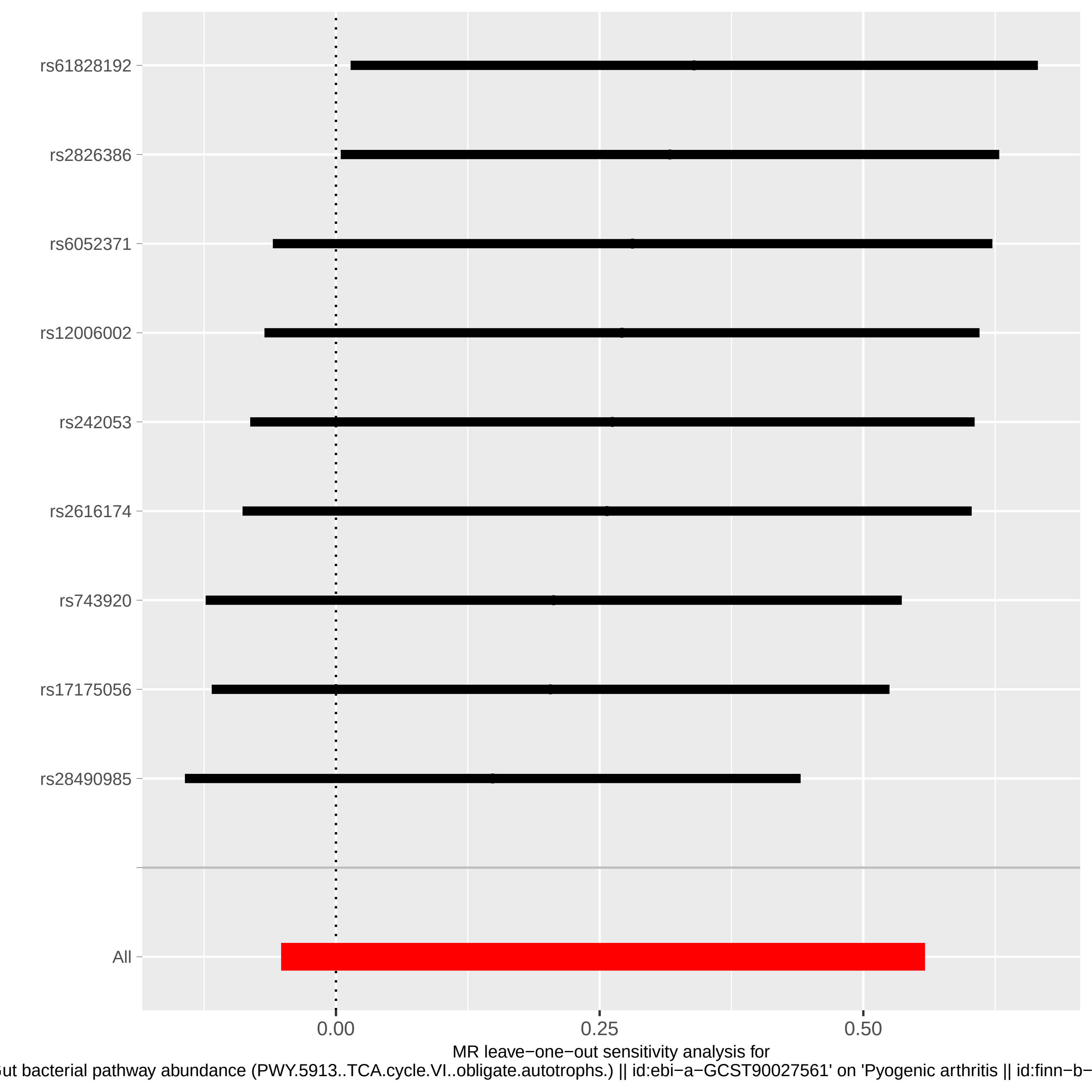
rs61828192
rs2826386
rs6052371
rs12006002
rs242053
rs2616174
rs743920
rs17175056
rs28490985
All
0.00
0.25
0.50
MR leave−one−out sensitivity analysis for
Gut bacterial pathway abundance (PWY.5913..TCA.cycle.VI..obligate.autotrophs.) || id:ebi−a−GCST90027561' on 'Pyogenic arthritis || id:finn−b−
